# [Retracted] MicroRNA‑195 suppresses rectal cancer growth and metastasis via regulation of the PI3K/AKT signaling pathway

**DOI:** 10.3892/mmr.2025.13543

**Published:** 2025-04-23

**Authors:** Yeli Wang, Linsong Mu, Miaoling Huang

Mol Med Rep 20: 4449–4458, 2019; DOI: 10.3892/mmr.2019.10717

Following the publication of the above paper, it was drawn to the Editor's attention by a concerned reader that, regarding the scratch wound assay experiments shown in Fig. 2A and the cell invasion assay experiments shown in Figs. 2B and 7A, a large number of data panels showed evidence of overlapping data, both within the same figure parts and comparing between Figs. 2 and 7.

Owing to the large number of data duplication events that have been identified in this paper, the Editor of *Molecular Medicine Reports* has decided that it should be retracted from the Journal on account of a lack of confidence in the presented data. The authors were asked for an explanation to account for these concerns, but the Editorial Office did not receive a reply. The Editor apologizes to the readership for any inconvenience caused.

